# Differential predictors for alcohol use in adolescents as a function of familial risk

**DOI:** 10.1038/s41398-021-01260-7

**Published:** 2021-03-04

**Authors:** Mira Tschorn, Robert C. Lorenz, Paul F. O’Reilly, Abraham Reichenberg, Tobias Banaschewski, Arun L. W. Bokde, Erin B. Quinlan, Sylvane Desrivières, Herta Flor, Antoine Grigis, Hugh Garavan, Penny Gowland, Bernd Ittermann, Jean-Luc Martinot, Eric Artiges, Frauke Nees, Dimitri Papadopoulos Orfanos, Luise Poustka, Sabina Millenet, Juliane H. Fröhner, Michael N. Smolka, Henrik Walter, Robert Whelan, Gunter Schumann, Andreas Heinz, Michael A. Rapp, IMAGEN Consortium, Trevor Robbins, Jeffrey Dalley, Naresh Subramaniam, David Theobald, Karl Mann, Christiane Bach, Maren Struve, Tobias Banaschewski, Herta Flor, Marcella Rietschel, Rainer Spanagel, Frauke Nees, Mira Fauth-Bühler, Sabina Millenet, Yvonne Grimmer, Luise Poustka, Mark Lathrop, Andreas Heinz, Lisa Albrecht, Nikolay Ivanov, Nicole Strache, Michael Rapp, Andreas Ströhle, Jan Reuter, Jürgen Gallinat, Henrik Walter, Isabel Gemmeke, Alexander Genauck, Caroline Parchetka, Katharina Weiß, Johann Kruschwitz, Bianca Raffaelli, Alev Isci, Laura Daedelow, Alexis Barbot, Benjamin Thyreau, Yannick Schwartz, Christophe Lalanne, Vincent Frouin, Dimitri Papadopoulos Orfanos, Antoine Grigis, John Rogers, James Ireland, Dirk Lanzerath, Jianfeng Feng, Jean-Luc Martinot, Zuleima Bricaud, Fanny Gollier Briand, Hervé LemaÎtre, Ruben Miranda, Eric Artiges, Jessica Massicotte, Marie-Laure Paillère Martinot, Helene Vulser, Jani Pentillä, Irina Filippi, André Galinowski, Pauline Bezivin, Gunter Schumann, Anna Cattrell, Tianye Jia, Sylvane Desrivières, Helen Werts, Lauren Topper, Laurence Reed, Chris Andrew, Catherine Mallik, Barbara Ruggeri, Charlotte Nymberg, Gareth Barker, Patricia J. Conrod, Lindsay Smith, Eva Loth, Stephanie Havatzias, Sheyda Shekarrizi, Emily Kitson, Alice Robinson, Deborah Hall, Chiara Rubino, Hannah Wright, Kerstin Stueber, Eanna Hanratty, Eleanor Kennedy, Fabiana Mesquita de Carvahlo, Argyris Stringaris, Gabriel Robert, Alex Ing, Christine Macare, Bing Xu, Tao Yu, Erin Burke Quinlan, Patrick Constant, Semiha Aydin, Ruediger Brühl, Albrecht Ihlenfeld, Bernadeta Walaszek, Bernd Ittermann, Michael Smolka, Thomas Hübner, Kathrin Müller, Stephan Ripke, Sarah Jurk, Eva Mennigen, Dirk Schmidt, Nora Vetter, Veronika Ziesch, Juliane H. Fröhner, Hugh Garavan, Arun L. W. Bokde, Robert Whelan, Daniel Carter, Emily Walsh, Susanne O’Driscoll, Maria Leonora Fatimah Agan, Mairead McMorrow, Sinead Nugent, Colm Connolly, Eoin Dooley, Clodagh Cremen, Jennifer Jones, John O’Keefe, Martin O’Connor, Jean-Baptiste Poline, Christian Büchel, Uli Bromberg, Tahmine Fadai, Juliana Yacubian, Sophia Schneider, Maria Lobatchewa, Claire Lawrence, Craig Newman, Kay Head, Nadja Heym, Penny Gowland, Alicia Stedman, Mehri Kaviani, Susannah Taplin, Dai Stephens, Tomáš Paus, Zdenka Pausova, Amir Tahmasebi

**Affiliations:** 1 Social and Preventive Medicine, Department of Sports and Health Sciences, Intra-faculty unit “Cognitive Sciences”, Faculty of Human Science, and Faculty of Health Sciences Brandenburg, Research Area Services Research and e-Health, University of Potsdam, Potsdam, Germany; 2 Social, Genetic and Developmental Psychiatry Centre, Institute of Psychiatry, Psychology and Neuroscience, King’s College London, London, UK; 3 Department of Genetics and Genomic Sciences, Icahn School of Medicine at Mount Sinai, New York, NY USA; 4 Department of Psychiatry, Icahn School of Medicine at Mount Sinai, New York, NY USA; 5 Department of Child and Adolescent Psychiatry and Psychotherapy, Central Institute of Mental Health, Medical Faculty Mannheim, Heidelberg University, Square J5, 68159 Mannheim, Germany; 6 Discipline of Psychiatry, School of Medicine and Trinity College Institute of Neuroscience, Trinity College Dublin, Dublin, Ireland; 7 Centre for Population Neuroscience and Precision Medicine (PONS), Institute of Psychiatry, Psychology & Neuroscience, SGDP Centre, King’s College London, London, UK; 8 Institute of Cognitive and Clinical Neuroscience, Central Institute of Mental Health, Medical Faculty Mannheim, Heidelberg University, Square J5, Mannheim, Germany; 9 Department of Psychology, School of Social Sciences, University of Mannheim, 68131 Mannheim, Germany; 10 NeuroSpin, CEA, Université Paris-Saclay, F-91191 Gif-sur-Yvette, France; 11 Departments of Psychiatry and Psychology, University of Vermont, 05405 Burlington, VT USA; 12 Sir Peter Mansfield Imaging Centre School of Physics and Astronomy, University of Nottingham, University Park, Nottingham, UK; 13 Physikalisch-Technische Bundesanstalt (PTB), Braunschweig and Berlin, Berlin, Germany; 14 Institut National de la Santé et de la Recherche Médicale, INSERM U1299 “Trajectoires développementales & psychiatrie”, University Paris Saclay, Ecole Normale supérieure Paris-Saclay, CNRS, Centre Borelli, Gif-sur-Yvette, France; 15 Institut National de la Santé et de la Recherche Médicale, INSERM U1299 “Trajectoires développementales & psychiatrie”, University Paris Saclay, Ecole Normale supérieure Paris-Saclay, CNRS, Centre Borelli; and Psychiatry Department, EPS Barthélémy Durand, Etampes, France; 16 Institute of Medical Psychology and Medical Sociology, University Medical Center Schleswig Holstein, Kiel University, Kiel, Germany; 17 Department of Child and Adolescent Psychiatry and Psychotherapy, University Medical Centre Göttingen, von-Siebold-Str. 5, 37075 Göttingen, Germany; 18 Department of Psychiatry and Neuroimaging Center, Technische Universität Dresden, Dresden, Germany; 19 Department of Psychiatry and Psychotherapy CCM, Charité – Universitätsmedizin Berlin, corporate member of Freie Universität Berlin, Humboldt-Universität zu Berlin, and Berlin Institute of Health, Berlin, Germany; 20 School of Psychology and Global Brain Health Institute, Trinity College Dublin, Dublin, Ireland; 21 PONS Research Group, Dept of Psychiatry and Psychotherapy, Campus Charite Mitte, Humboldt University, Berlin and Leibniz Institute for Neurobiology, Magdeburg, Germany, and Institute for Science and Technology of Brain-inspired Intelligence (ISTBI), Fudan University, Shanghai, PR China; 22 Behavioural and Clinical Neuroscience Institute, Cambridge University, Cambridge, UK; 23 Central Institute of Mental Health, Mannheim, Germany; 24 Centre National de Génotypage, Genoscope, Èvry, France; 25 Comissariat à l’Energie Atomique, Fontenay-aux-Roses, France; 26 Delosis, Twickenham, UK; 27 Deutsches Referenzzentrum für Ethik, Bonn, Germany; 28 Fudan University, Shanghai, China and Warwick University, Coventry, UK; 29 Group d’Imagerie Neurofonctionelle, Institut de Maladies Neurodégénératives, CNRS UMR 5293, Université de Bordeaux, Centre Broca Nouvelle-Aquitaine, Bordeaux, France; 30 Institut National de la Santé et de la Recherche Médicale, INSERM U1299 “Trajectoires développementales & psychiatrie”, University Paris Saclay, Ecole Normale supérieure Paris-Saclay, CNRS, Centre Borelli and AP-HP.Sorbonne Université, Department of Child and Adolescent Psychiatry, Pitié-Salpêtrière Hospital, Paris, France; 31 Pertimm, Paris, France; 32 Trinity College Dublin, Dublin, Ireland; 33 University College Dublin, Dublin, Ireland; 34 McGill University, Montréal, Canada and University of California, Berkeley, USA; 35 University Medical Centre Hamburg-Eppendorf, Hamburg, Germany; 36 University of Nottingham, University Park, Nottingham, UK; 37 University of Sussex, Brighton, UK; 38 University of Toronto, Toronto, ON Canada

**Keywords:** Genetics, Neuroscience, Psychology, Addiction

## Abstract

Traditional models of future alcohol use in adolescents have used variable-centered approaches, predicting alcohol use from a set of variables across entire samples or populations. Following the proposition that predictive factors may vary in adolescents as a function of family history, we used a two-pronged approach by first defining clusters of familial risk, followed by prediction analyses within each cluster. Thus, for the first time in adolescents, we tested whether adolescents with a family history of drug abuse exhibit a set of predictors different from adolescents without a family history. We apply this approach to a genetic risk score and individual differences in personality, cognition, behavior (risk-taking and discounting) substance use behavior at age 14, life events, and functional brain imaging, to predict scores on the alcohol use disorders identification test (AUDIT) at age 14 and 16 in a sample of adolescents (*N* = 1659 at baseline, *N* = 1327 at follow-up) from the IMAGEN cohort, a longitudinal community-based cohort of adolescents. In the absence of familial risk (*n* = 616), individual differences in baseline drinking, personality measures (extraversion, negative thinking), discounting behaviors, life events, and ventral striatal activation during reward anticipation were significantly associated with future AUDIT scores, while the overall model explained 22% of the variance in future AUDIT. In the presence of familial risk (*n* = 711), drinking behavior at age 14, personality measures (extraversion, impulsivity), behavioral risk-taking, and life events were significantly associated with future AUDIT scores, explaining 20.1% of the overall variance. Results suggest that individual differences in personality, cognition, life events, brain function, and drinking behavior contribute differentially to the prediction of future alcohol misuse. This approach may inform more individualized preventive interventions.

## Introduction

Alcohol use disorder (AUD) is a complex psychiatric condition that involves both genetic and environmental factors. In adolescents, alcohol use is common with 10% of 14-year-olds in the National Comorbidity Survey reporting regular alcohol use, and up to 27% of the American 16-year-olds reporting regular alcohol use^1^ and up to 12% suffering from any type of substance use disorder^2^. In Europe, estimates of alcohol misuse (specifically, binge drinking) have been as high as 59% for 30-day prevalence rates for 15–16-year-olds^3^. The development of AUD in adolescence is one strong predictor for future (adult) alcohol dependence^4^ which cannot be fully explained by genetics^5^.

Beyond early alcohol use^4,5^ the most consistently identified risk factors for adolescent alcohol misuse include childhood adversity and other negative life events^6^, cognitive dysfunction and impulsivity^7–9^, as well as early (peer) deviance^10,11^. Genetic association studies have pointed to polygenetic risk scores^12,13^. Additionally, a significant contribution of familial risk to adolescent alcohol misuse has long been proposed^14–18^. Parental AUD was shown to predict the onset of hazardous alcohol use and alcohol dependence in adolescents but not non-problematic drinking^18^. Furthermore, next to genetic factors, neurobiological reward-related mechanisms have been proposed as a possible link between familial risk and alcohol misuse, but empirical results showed that adolescents with familial risk of alcohol misuse compared to adolescents without familial risk showed no difference in reward-related activation in the ventral striatum (VS)^19^. However, reward system activity was increased in adult participants with a AUD family history as compared to adults without family history^20^.

Recently, longitudinal analyses of incipient AUD in adolescence have used more elaborate designs and/or analyses to improve both the predictive power and causal understanding of AUD development in adolescents. For example, Kendler et al. have used different groupings of longitudinal trajectories of consumption patterns to study associations to genetic and behavioral risk factors in adolescence^11,21,22^. Whelan et al. have used machine learning approaches to validate neuropsychological profiles of future alcohol misuse including individual differences in personality and cognition, substance use at age 14, life events, and functional brain imaging^23^, yielding predictive validity with precision rates as high as 91%.

Nees et al. were able to explain 17% of variance in early onset of drinking in 14-year-old adolescents with a latent factor for brain regions (0.4% variance), personality (16% variance), and behavior (0.6% variance)^8^ in data from the same cohort as the present study (IMAGEN). Parental alcohol use, however, did not predict early onset of drinking, therefore a role of parental AUD only at later stages in adolescents was suggested. This finding is in line with other findings, also from the IMAGEN cohort, where candidate genetic variation only significantly added to predicting alcohol drinking behavior in 16-year-old adolescents, but not in 14-year-olds^24^. In early adolescence (14 years) personality traits contributed to the main part of the 13% explained variance of alcohol drinking behavior, whereas genetic variations, reward-related brain response, and behavior did not significantly predict adolescent alcohol drinking. At age 16, personality and genetic variation contributed to the 14% explained variance in alcohol drinking.

Despite the precision achieved in prediction with large amounts of variance explained using different sets of longitudinal trajectories, the current approaches make it difficult to predict and identify risk factors at an individual or even subgroup level, since they either employ sets of variables across cohorts (i.e., are variable-centered^23^) or rely on future change of predictors themselves^11,22^.

Previous studies in young adults have shown that patients with a family history of drug abuse may exhibit different sets of predictors for alcohol and drug abuse as compared to adults without a family history of drug abuse^25^. In adults with a positive family history of drug abuse, especially impulsivity and risk-taking behaviors have recently been associated with increased and harmful alcohol use^26^. This relationship between impulsivity and family history is further supported by genetic analyses that propose impulsivity to be a mechanistic mediator of familial risk for alcohol abuse^27^. At the same time, in a prior analysis from the IMAGEN study, there was no significant difference in ventral striatal activity in adolescents with and without a family history of substance abuse, suggesting that alterations in the reward system could be independent of familial risk factors^19^. Therefore, there is a likelihood that different predictor sets for alcohol use are at work in adolescents with as compared to without a family history of substance abuse. Here, we specifically tested for the first time whether adolescents with a family history of drug abuse exhibit a set of predictors different from adolescents without a family history. Such patterns would not necessarily imply that family history itself acts as a potent risk factor in early adolescence, but that the effects of predictors may vary as a function of family history.

One strategy to test for such effects would be to stratify adolescents in groups according to baseline variables, e.g. using latent class or other clustering approaches. Such an approach would allow to test different predictive models in different groups, i.e. hypotheses that risk profiles may differ between groups of adolescents based on a predefined stratification. In the present study, our primary aim was to test the feasibility and predictive utility of such an approach using data from the IMAGEN cohort^23,28^. Given the potentially numerous interactions between genetic and familial risk factors on the one hand, and behavioral risk factors on the other, we decided to a priori cluster subjects according to familial risk, and then test for differences in predictive models within these familial risk groups. While we could not state a clear a-priori hypothesis based on interactions between genetic and behavioral factors, we tentatively hypothesized that baseline drinking, neurobiological alterations of the reward system and candidate genetic risk would be salient predictors of future alcohol abuse in adolescents with familial risk, while individual differences in personality would be more predictive in adolescents without familial risk.

Thus, in the present study, we aimed to characterize differential predictors of hazardous alcohol use in 16-year-old adolescents with and without familial risk.

## Methods

### Sample

We used a sample of 2240 adolescents from the multicenter IMAGEN study^28^ with available data from neuropsychological, imaging, and genetic assessments (see Table 1). School-based recruitment at age 14 took place at eight different sites in Germany, the United Kingdom, France, and Ireland. Subjects ineligible for MRI-Scans were excluded as well as adolescents suffering from serious medical conditions (e.g. diabetes, rheumatologic diseases, neurological disorders, or developmental conditions). We used data from the first and second waves of IMAGEN. A detailed description of the recruitment and assessment procedures has been published elsewhere^28^. All local ethics research committees approved the study in accordance with the Declaration of Helsinki. Written informed consent was obtained from the parent or guardian, and verbal assent was obtained from the adolescent.

**Table 1:** Demographic and clinical characteristics.

Baseline sample	Overall (*N* = 1659)	Familial risk (*N* = 866)	No familial risk (*N* = 793)	*p*
Female *N* (%)	838 (50.5)	412 (47.6)	426 (53.7)	0.037
Age *M* ± *SD*	14.55 ± 0.43	14.52 ± 0.42	14.58 ± 0.44	0.004
SES *M* ± *SD*	17.86 ± 3.88	18.04 ± 3.87	17.66 ± 3.89	0.050
AUDIT baseline *M* ± *SD*	1.48 ± 2.52	1.62 ± 2.82	1.33 ± 2.12	0.022
Baseline drinking *M* ± *SD*	2.00 ± 1.72	2.06 ± 1.71	1.94 ± 1.72	0.170
Baseline smoking *M* ± *SD*	0.80 ± 1.62	0.75 ± 1.59	0.86 ± 1.66	0.156
FH alcohol *median* ± *SD* FH alcohol negative *N* (%)	1.00 ± 0.57 793 (47.8)	1.00 ± 0.26 0 (0)	0.00 ± 0.00 793 (100)	0.000 0.000
FH drug *median* ± *SD* FH drug negative *N* (%)	0.00 ± 0.51 908 (54.7)	1.00 ± 0.36 115 (13.3)	0.00 ± 0.00 793 (100)	0.000 0.000

Follow-up sample	Overall (*N* = 1327)	Familial risk (*N* = 711)	No familial risk (*N* = 616)	*p*
Female *N* (%)	690 (52.0)	348 (48.9)	342 (55.5)	0.017
Age *M* ± *SD*	16.48 ± 0.62	16.46 ± 0.64	16.51 ± 0.59	0.119
SES *M* ± *SD*	18.07 ± 3.72	18.20 ± 3.72	17.92 ± 3.73	0.173
AUDIT FU *M* ± *SD*	3.81 ± 3.47	4.03 ± 3.59	3.55 ± 3.31	0.011

*SES* socioeconomic status, *AUDIT* Alcohol Use Disorder Identification Test, *FH* family history, *FU* follow up, *M* mean, *SD* standard deviation.

### Measures

#### Functional imaging tasks

The IMAGEN task-related fMRI datasets were reanalyzed at the Neurospin (Paris). We used these reanalyzed datasets for functional imaging tasks.

Subjects were scanned in 3T-MRI-Scanners from different manufacturers (Bruker, General Electric, Philips, and Siemens, see^28^). Functional data was acquired using a gradient-echo-planar-imaging (epi) T2*-weighted sequence (echo time 30 ms, repetition time 2.2 s, flip angle 75°). 300 volumes for MID task and 444 volumes for Stop Signal Task (SST) (see below) were obtained, each consisting of 40 slices (2.4 mm thickness. 1 mm gap, voxel size: 3.4 mm × 3.4 mm × 3.4 mm). Analyses were performed using SPM12 (Wellcome Trust Centre for Neuroimaging). Individual fMRI-images were slice-time corrected, spatially realigned, and normalized on the Montreal Neurological Institute space using a custom epi template, which was created on the mean of a 200 subjects set. Finally, images were smoothed with a 5-mm Gaussian filter.

For the first-level analyses, a general linear model was individually computed with a design-matrix including the experimental regressors (see below) as well as 21 additional movement regressors (3 translations, 3 rotations, 3 translations shifted 1 TR before, 3 translations shifted 1 TR later, and 9 additional regressors corresponding to the long term effects of the movement).

##### Monetary Incentive Delay (MID)

The Monetary Incentive Delay (MID) task^29^ required participants to respond after seeing a cue (250 ms) and a delay of 4–4.5 s (blank screen) to a briefly presented target (250–400 ms) by pressing either a left-hand or right-hand button as quickly as possible to indicating monitor appearing side. Participants scored points when responding while the target was on the screen, whereas they did not receive points in case they responded after disappearing of the target. A trial onset cue reliably indicated target position and gain condition. A triangle indicated no points, a one-lined circle 2 points and a three-lined circle 10 points. For the first-level analysis, experimental events were modeled by convolving the canonical hemodynamic response function with the onsets of the anticipation and feedback (hit or miss) periods for each cue and feedback type as well as button presses. Individual contrast images were calculated for anticipation (large gain versus small gain) and feedback phase (large gain versus no gain) in hit trials. On the second level, these differential t-contrast images were entered to one sample *t*-tests including scanning site as covariate. Regions of interest (ROI) analyses were conducted using literature-based ROIs of the functional key nodes VS, insula, and ventromedial prefrontal cortex (VMPFC)^30^.

##### Stop Signal Task (SST)

The Stop Signal Task (SST^31^) required participants to respond to visual go stimuli (left or right arrow) but to withhold their motor response when the go stimulus was followed unpredictably by a stop signal (upward arrow). Task difficulty was individually adjusted using an algorithm which has been described elsewhere^32^. The SST contained 400 go trials with a stimulus duration of 1000 ms and 80 stop trials with a stimulus duration of 0–900 ms in accordance to the algorithm.

First-level analysis was conducted with the experimental regressors stop successful, stop failure, and two types of failures (button press too late and wrong direction). These events were modeled by convolving the canonical hemodynamic response function with the onsets of the trial types. Consequently, individual differential t-contrast images were conducted for successful stop trials versus unsuccessful stop trials and taken to second-level analysis. These t-contrast images were used for one sample *t*-tests including scanning site as covariate. ROI analyses were conducted for orbitofrontal cortex and right inferior frontal gyrus (pars triangularis) as described in the Anatomic Labeling brain atlas^33^. These ROI were of particular interest, because previous research showed an enhanced importance of these regions in adolescence illicit substance use^34^.

#### Personality measures

Three instruments were used to assess personality: the dimension extraversion of the 60-item neuroticism-extraversion-openness five-factor inventory (NEO-PI-R^35^); the hopelessness dimension from the Substance Use Risk Profile Scale (SURPS), which assesses personality traits that confer risk for substance misuse and psychopathology^36^; impulsiveness was measured via the revised Temperament and Character Inventory (TCI-R^37^).

#### Cognition and behavior

Participants completed of the Wechsler intelligence scale for children WISC-IV, of which we included two indices: perceptual reasoning index (PRI: block design, picture concepts, matrix reasoning) and verbal comprehension index (VRI: similarities, vocabulary, information, comprehension)^38^.

Delay discounting as the preference of smaller immediate over delayed larger rewards was measured using the Monetary-Choice Questionnaire^39^.

Participants completed a slightly modified version of the Cambridge Gambling Task (CGT) from the Cambridge Cognition Neuropsychological Test Automated Battery for a measurement of risk-taking outside a learning context.

#### Stressful life events

The life-events questionnaire (LEQ^40^) uses 39 items to measure the lifetime occurrence and the perceived desirability of stressful events. We included a sum score of two domains that are relevant for vulnerability and prediction of substance abuse: sexuality and deviance.

#### Demographics

The socioeconomic status score comprised the sum of the following domains: parents’ education, family stress, unemployment, financial difficulties, home inadequacy, neighborhood, financial crisis, parents’ employment.

#### Genetics

We included single nucleotide polymorphisms (SNPs) described in a review and a genome-wide association study of alcohol dependence^13,28^. Of the 30 SNPs listed in that review, the IMAGEN sample data contained 15 SNPS that passed quality control, did not have a low minor allele frequency (<5%) or a high genotyping failure rate (>5%), and were not highly correlated (>0.21) with any other available SNP. Genetic data were available on 1835 individuals. From these data, we calculated a genetic risk score (SNP risk score) by summing up the 15 trait-associated alleles across many genetic loci, weighted by effect sizes estimated from a genome-wide association study^41^.

#### Substance misuse measures

The European School Survey Project on Alcohol and Drugs battery (ESPAD) was administered^42^. The primary questions of interest were regarding lifetime alcohol use and lifetime cigarette consumption. In addition, at baseline (age 14) as well as at follow-up (age 16), we used the alcohol use disorders identification test (AUDIT^43^; self-report version) score. The AUDIT is a 10-item screening tool to assess alcohol consumption, drinking behaviors, and alcohol-related problems. A score of 8 or more is considered to indicate hazardous alcohol use.

#### Familial risk of substance misuse

Familial risk of drug and alcohol misuse was derived from multiple measurements and categorized in “positive family history” (score 2), “negative family history” (score 0), and “intermediate family history” (score 1, neither positive nor negative, see also^19,23^). To assess familial risk of illicit drug and alcohol misuse, the following measurements were used: the Michigan Alcohol Screening Test (MAST^44^), a family history interview on substance misuse, parent-administered AUDIT and the Drug Abuse Screening Test (DAST^45^). An intermediate family history of alcohol misuse or illicit drug use was classified when parents showed elevated scores on MAST, DAST, or AUDIT without clear indication for misuse or when alcohol or illicit drug misuse was assessed for second degree relatives. An intermediate family history of illicit drug misuse was identified, when parents scored higher on DAST or drug misuse was assessed for second degree relatives or when family history of alcohol misuse was positive.

Participant’s parents completed the Pregnancy and Birth Questionnaire (PBQ, adapted from^46^) as self-report measurement on gestational alcohol and cigarette exposure. Scores were recoded into binary variables.

### Data analysis

All analyses were performed using SPSS version 25. All tests were performed two-sided.

#### Two-step Clustering

To group adolescents regarding their family history of substance abuse, we conducted a Two-Step Cluster analysis (TSC) using family history of substance misuse (alcohol and illicit drugs) as input variables. We compared the resulting clusters regarding the family history variables by conducting Mann–Whitney *U* tests. This data reduction approach was implemented in order to separate participants according to familial risk, assessed by two variables á three categories.

#### Multiple regression

We performed collinearity diagnostics for all regression models by analyzing the Variance Inflation Factors and the tolerance statistics, which did not reveal any multicollinearity in the reported models. Since there was evidence for heteroskedasticity in AUDIT baseline scores, we compared results from baseline regression models with heteroscedasticity-consistent parameters for those models.

##### Overall sample

We conducted two multiple regression analyses to predict hazardous drinking (AUDIT) at baseline (14 years) and follow-up (16 years) in the overall sample. Twenty-eight predictors were pre-defined as described above.

##### AUDIT baseline (14 years)

We conducted two separate regression models to predict hazardous drinking at baseline in two clusters using Bonferroni adjusted α-levels of 0.025 to control for multiple testing.

##### AUDIT follow-up (16 years)

Analogously, we conducted two separate regression models to predict hazardous drinking at follow-up in two clusters and adjusted α-levels to 0.025.

## Results

### Two-step Clustering

The overall sample used for two-step cluster analysis (*N* = 2240) comprised 50.4% female adolescents, with mean age of 14.55 years (*SD* = 0.43). The two-step cluster analysis revealed two clusters on the basis of two variables for familial risk for alcohol and illicit drug misuse (cluster 1*N* = 1187, cluster 2*N* = 1048, and one outlier cluster *N* = 5). Full data on other demographic variables and defined predictors was available for a total *N* = 1659 at baseline and *N* = 1327 at follow-up. Characteristics of all subjects and clusters are shown in Table 1. Clusters clearly separated according to substance misuse history in that cluster 1 was characterized by a presence of familial risk for alcohol or drug misuse (intermediate to positive family history of drug or alcohol misuse), whereas participants in Cluster 2 showed no such family history (negative family history of alcohol and drug misuse). Both variables for familial risk differed significantly between the two clusters (FH-drug: *p* = 0.000, FH-alcohol: *p* = 0.000). The silhouette coefficient of 0.9 indicates a good cohesion and cluster separation. The ratio of cluster sizes is 1.13 and therefore below 3.0, suggesting a satisfactory cluster solution.

### Predicting hazardous drinking

#### Overall sample

As detailed in Table 2, in our overall sample (*N* = 1659 14-year-old adolescents) higher scores of hazardous drinking (AUDIT) were predicted by higher occurrence of sexual and deviant life events (LEQ, *β* = 0.119, *CI* = 0.071–0.167, *p* = 0.000), more hopelessness (SURPS, *β* = 0.104, *CI* = 0.057–0.151, *p* = 0.000), more extraversion (NEO, *β* = 0.067, *CI* = 0.020–0.114, *p* = 0.006), more smoking (ESPAD lifetime use, *β* = 0.385, *CI* = 0.338–0.432, *p* = 0.000), familial risk of alcohol and drug misuse (*β* = −0.050, *CI* = −0.093 to −0.007, *p* = 0.024), and higher prenatal alcohol exposure (PBQ, *β* = 0.079, *CI* = 0.035–0.123, *p* = 0.000). Heteroscedasticity-consistent parameters revealed identical significant predictors.

**Table 2:** Prediction of AUDIT at baseline and follow-up in the overall sample.

	AUDIT baseline (14 years) *N* = 1659			AUDIT follow-up (16 years) *N* = 1327		
Variables	Standardized beta		*p*	Standardized beta		*p*
LEQ (sexuality, deviance)	0.119	(0.071–0.167)	0.000	0.131	(0.075–0.187)	0.000
SURPS hopelessness	0.103	(0.057–0.151)	0.000	0.061	(0.007–0.115)	0.026
MCQ discounting	0.030	(−0.014–0.074)	0.182	−0.035	(−0.085–0.015)	0.169
TCI impulsivity	0.019	(−0.027–0.065)	0.420	0.062	(0.010–0.114)	0.021
NEO extraversion	0.067	(0.020–0.114)	0.006	0.124	(0.069–0.179)	0.000
WISC-IV PRI	−0.035	(−0.088–0.012)	0.161	−0.025	(−0.083–0.033)	0.380
WISC-IV VCI	0.014	(−0.042–0.077)	0.584	0.031	(−0.023–0.085)	0.282
CGT risk-taking	−0.002	(−0.042–0.038)	0.923	0.053	(0.004–0.102)	0.035
SNP risk score	0.003	(−0.041–0.047)	0.894	−0.021	(−0.071–0.029)	0.408
SES	−0.003	(−0.048–0.042)	0.905	0.012	(−0.043–0.068)	0.655
Baseline smoking	0.385	(0.338–0.432)	0.000	0.062	(0.006–0.118)	0.032
Baseline drinking	–		–	0.258	(0.203–0.314)	0.000
FH alcohol and drugs	−0.050	(−0.093 to −0.007)	0.024	−0.058	(−0.098 to −0.002)	0.059
MID Insula anticipation	−0.015	(−0.064–0.033)	0.543	−0.027	(−0.083–0.029)	0.344
MID VS anticipation	0.007	(−0.048–0.062)	0.805	0.082	(0.020–0.144)	0.010
MID VMPFC anticipation	0.008	(−0.039–0.055)	0.742	−0.086	(−0.141 to −0.031)	0.002
MID Insula outcome	0.019	(−0.029–0.068)	0.439	−0.057	(−0.112 to −0.002)	0.042
MID VS outcome	−0.032	(−0.082–0.019)	0.217	0.079	(0.020–0.138)	0.009
MID VMPFC outcome	−0.038	(−0.085–0.009)	0.118	−0.020	(−0.074–0.034)	0.466
SST OFC	−0.004	(−0.068–0.060)	0.901	0.008	(−0.058–0.074)	0.811
SST rIFG	−0.031	(−0.089–0.027)	0.298	0.022	(−0.047–0.091)	0.535
Prenatal cigarette consumption	0.008	(−0.035–0.051)	0.714	0.000	(−0.055–0.054)	0.986
Prenatal alcohol consumption	0.079	(0.035–0.123)	0.000	0.006	(−0.050–0.062)	0.834

*AUDIT* Alcohol Use Disorder Identification Test, *LEQ* Life Events Questionnaire, *SURPS* Substance Use Risk Profile Scale, *TCI* Temperament and Character Inventory, *NEO* Revised NEO Personality Inventory—NEO PI-R, *WISC-IV* Wechsler Intelligence Scale for Children—Fourth Edition, *PRI* perceptual reasoning index, *VCI* verbal comprehension index, *CGT* Cambridge Gambling Task, *SNP* single nucleotide polymorphisms, *SES* socioeconomic status, *FH* family history, *MID* Monetary Incentive Delay, *VS* ventral striatum, *VMPFC* ventromedial prefrontal cortex, *SST* Stop Signal Task, *OFC* orbitofrontal cortex, *rIFG* right inferior frontal gyrus, AUDIT baseline *R*^*2*^ = 0.244, AUDIT follow-up *R*^*2*^ = 0.193.

Higher scores of hazardous drinking (AUDIT) 2 years later (*N* = 1376 16-year-old adolescents) were predicted by higher occurrence of sexual and deviant life events (LEQ, *β* = 0.131, *CI* = 0.075–0.187, *p* = 0.000), more hopelessness (SURPS, *β* = 0.061, *CI* = 0.007–0.115, *p* = 0.026), stronger impulsivity (TCI, *β* = 0.062, *CI* = 0.010–0.114, *p* = 0.021), more extraversion (NEO, *β* = 0.124, *CI* = 0.069–0.179, *p* = 0.000), riskier behavior (CGT, *β* = 0.053, *CI* = 0.004–0.102, *p* = 035), more smoking and drinking at baseline (ESPAD lifetime use, *β* = 0.062, *CI* = 0.006–0.118, *p* = 0.032; *β* = 0.258, *CI* = 0.203–0.314, *p* = 0.000), stronger ventral striatal activation during reward anticipation (MID, *β* = 0.082, *CI* = 0.020–0.144, *p* = 0.010) and outcome processing (MID, *β* = 0.079, *CI* = 0.020–0.138, *p* = 0.009), weaker ventromedial prefrontal activation during reward anticipation (MID, *β* = −0.086, *CI* = −0.141 to −0.031, *p* = 0.002), and weaker insula activation during outcome processing (MID, *β* = −0.057, *CI* = −0.112 to −0.002, *p* = 0.042).

#### Prediction of AUDIT scores at baseline as a function of familial risk

As detailed in Table 3, higher scores of hazardous drinking in 14-year-old adolescents with familial risk for alcohol and drug abuse (*N* = 866) were predicted by higher occurrence of sexual and deviant life events (LEQ, *β* = 0.153, *CI* = 0.086–0.219, *p* = 0.000), more hopelessness (SURPS, *β* = 0.100, *CI* = 0.033–0.167, *p* = 0.003), and more frequent lifetime smoking (ESPAD, *β* = 0.336, *CI* = 0.271–0.401, *p* = 0.000). Heteroscedasticity-consistent parameters additionally revealed extraversion (*p* = 0.014) and prenatal alcohol consumption (*p* = 0.023) as significant predictors of hazardous drinking.

**Table 3:** Prediction of AUDIT scores at baseline as a function of familial risk.

	Familial risk *N* = 866			No familial risk *N* = 793		
Variables	Standardized beta		*p*	Standardized beta		*p*
LEQ (sexuality, deviance)	0.153	(0.086–0.219)	0.000	0.068	(−0.001–0.137)	0.054
SURPS hopelessness	0.100	(0.033–0.167)	0.003	0.098	(0.034–0.162)	0.003
MCQ discounting	0.047	(−0.014–0.108)	0.134	0.015	(−0.046–0.076)	0.632
TCI impulsivity	0.003	(−0.053–0.057)	0.931	0.045	(−0.017–0.107)	0.160
NEO extraversion	0.073	(0.004–0.142)	0.038	0.063	(0.002–0.128)	0.057
WISC-IV PRI	−0.047	(−0.120–0.021)	0.177	−0.017	(−0.074–0.045)	0.617
WISC-IV VCI	0.002	(−0.058–0.058)	0.947	0.028	(−0.042–0.105)	0.419
CGT risk-taking	0.013	(−0.049–0.075)	0.682	−0.035	(−0.095–0.025)	0.252
SNP risk score	−0.003	(−0.066–0.060)	0.926	0.019	(−0.041–0.079)	0.535
SES	−0.010	(−0.069–0.050)	0.754	0.002	(−0.068–0.072)	0.948
Baseline smoking	0.336	(0.271–0.401)	0.000	0.477	(0.410–0.544)	0.000
MID Insula anticipation	−0.027	(−0.097–0.043)	0.449	−0.014	(−0.082–0.054)	0.686
MID VS anticipation	−0.051	(−0.127–0.025)	0.189	0.103	(0.028–0.178)	0.007
MID VMPFC anticipation	0.047	(−0.021–0.114)	0.172	−0.049	(−0.117–0.019)	0.157
MID Insula outcome	0.000	(−0.000–0.000)	0.998	0.048	(−0.015–0.111)	0.137
MID VS outcome	−0.056	(−0.132–0.020)	0.147	−0.001	(−0.046–0.045)	0.969
MID VMPFC outcome	0.003	(−0.051–0.057)	0.920	−0.093	(−0.160 to −0.027)	0.006
SST OFC	0.011	(−0.074–0.096)	0.799	−0.009	(−0.083–0.065)	0.814
SST rIFG	−0.023	(−0.106–0.060)	0.588	−0.057	(−0.136–0.022)	0.157
Prenatal cigarette consumption	0.018	(−0.046–0.082)	0.578	−0.029	(−0.080–0.042)	0.538
Prenatal alcohol consumption	0.069	(0.007–0.130)	0.028	0.098	(0.038–0.157)	0.001

*AUDIT* Alcohol Use Disorder Identification Test, *LEQ* Life Events Questionnaire, *SURPS* Substance Use Risk Profile Scale, *TCI* Temperament and Character Inventory, *NEO* Revised NEO Personality Inventory—NEO PI-R, *WISC-IV* Wechsler Intelligence Scale for Children—Fourth Edition, *PRI* perceptual reasoning index, *VCI* verbal comprehension index, *CGT* Cambridge Gambling Task, *SNP* single nucleotide polymorphisms, *SES* socioeconomic status, *FH* family history, *MID* Monetary Incentive Delay, *VS* ventral striatum, *VMPFC* ventromedial prefrontal cortex, *SST* Stop Signal Task, *OFC* orbitofrontal cortex, *rIFG* right inferior frontal gyrus, familial risk *R*^*2*^ = 0.224, no familial risk *R*^*2*^ = 0.315.

Higher scores of hazardous drinking in 14-year-old adolescents without familial risk of alcohol and drug abuse (*N* = 793) were predicted by more hopelessness (SURPS, *β* = 0.098, *CI* = 0.034–0.162, *p* = 0.003), more smoking (ESPAD, *β* = 0.477, *CI* = 0.410–0.544, *p* = 0.000), stronger ventral striatal activation during reward anticipation (MID, *β* = 0.103, *CI* = 0.028–0.174, *p* = 0.007), weaker ventromedial prefrontal activation during outcome processing (MID, *β* = −0.093, *CI* = −0.160 to −0.027, *p* = 0.006) and higher prenatal alcohol exposure (PBQ, *β* = 0.098, *CI* = 0.038–0.157, *p* = 0.001). Heteroscedasticity-consistent parameters revealed identical significant predictors.

#### Prediction of AUDIT scores at follow-up as a function of familial risk

Higher scores of hazardous drinking 2 years later in 16-year-old adolescents with familial risk for alcohol and drug abuse (*N* = 711, see Table 4) were predicted by higher occurrence of sexual and deviant life events (LEQ, *β* = 0.129, *CI* = 0.052–0.198, *p* = 0.001), stronger impulsivity (TCI, *β* = 0.098, *CI* = 0.025–0.174, *p* = 0.009), more extraversion (NEO, *β* = 0.117, *CI* = 0.039–0.189, *p* = 0.003), and more drinking at baseline (ESPAD, *β* = 0.234, *CI* = 0.158–0.311, *p* = 0.000).

**Table 4:** Prediction of AUDIT scores at follow-up as a function of familial risk.

	Familial risk *N* = 711			No familial risk *N* = 616		
Variables	Standardized beta		*p*	Standardized beta		*p*
LEQ (sexuality, deviance)	0.129	(0.052–0.198)	0.001	0.151	(0.067–0.236)	0.000
SURPS hopelessness	0.028	(−0.046–0.100)	0.461	0.090	(0.012–0.175)	0.024
MCQ discounting	0.006	(−0.066–0.077)	0.871	−0.083	(−0.1658 to −0.014)	0.031
TCI impulsivity	0.098	(0.025–0.174)	0.009	0.009	(−0.062–0.073)	0.812
NEO extraversion	0.117	(0.039–0.189)	0.003	0.123	(−0.044–0.206)	0.002
WISC-IV PRI	0.001	(−0.050–0.090)	0.970	−0.055	(−0.140–0.024)	0.186
WISC-IV VCI	0.034	(−0.045–0.108)	0.396	0.017	(−0.068–0.106)	0.674
CGT risk-taking	0.076	(0.007–0.–0.148)	0.030	0.035	(−0.038–0.113)	0.348
SNP risk score	−0.054	(−0.123–0.011)	0.123	0.022	(−0.049–0.098)	0.544
SES	0.031	(−0.044–0.110)	0.418	−0.007	(−0.077–0.072)	0.854
Baseline smoking	0.074	(−0.004–0.155)	0.061	0.050	(−0.036–0.141)	0.253
Baseline drinking	0.234	(0.158–0.311)	0.000	0.283	(0.200–0.359)	0.000
MID Insula anticipation	−0.026	(−0.105–0.053)	0.517	−0.030	(−0.114–0.054)	0.484
MID VS anticipation	0.046	(−0.043–0.–0.135)	0.311	0.132	(0.042–0.222)	0.004
MID VMPFC anticipation	−0.078	(−0.154 to−0.002)	0.044	−0.092	(−0.174 to −0.010)	0.028
MID Insula outcome	−0.062	(−0.140–0.016)	0.122	−0.047	(−0.126–0.032)	0.242
MID VS outcome	0.071	(−0.015–0.157)	0.105	0.082	(−0.002–0.166)	0.055
MID VMPFC outcome	0.001	(−0.051–0.053)	0.970	−0.034	(−0.114–0.046)	0.407
SST OFC	0.012	(−0.084–0.107)	0.896	−0.002	(−0.111–0.107)	0.973
SST rIFG	0.018	(−0.078–0.114)	0.713	0.026	(−0.073–0.125)	0.607
Prenatal cigarette consumption	−0.043	(−0.116–0.029)	0.287	0.050	(−0.027–0.127)	0.202
Prenatal alcohol consumption	0.018	(−0.051–0.087)	0.664	−0.003	(−0.077–0.071)	0.937

*AUDIT* Alcohol Use Disorder Identification Test, *LEQ* Life Events Questionnaire, *SURPS* Substance Use Risk Profile Scale, *TCI* Temperament and Character Inventory, *NEO* Revised NEO Personality Inventory—NEO PI-R, *WISC-IV* Wechsler Intelligence Scale for Children—Fourth Edition, *PRI* perceptual reasoning index, *VCI* verbal comprehension index, *CGT* Cambridge Gambling Task, *SNP* single nucleotide polymorphisms, *SES* socioeconomic status, *FH* family history, *MID* Monetary Incentive Delay, *VS* ventral striatum, *VMPFC* ventromedial prefrontal cortex, *SST* Stop Signal Task, *OF*C orbitofrontal cortex, *rIFG* right inferior frontal gyrus, familial risk *R*^*2*^ = 0.192, no familial risk *R*^*2*^ = 0.214.

Higher scores of hazardous drinking 2 years later in 16-year-old adolescents without familial risk for alcohol and drug abuse (*N* = 616) were predicted by higher occurrence of sexual and deviant life events (LEQ, *β* = 0.151, *CI* = 0.067–0.236, *p* = 0.000), more hopelessness (SURPS, *β* = 0.090, *CI* = 0.012–0.175, *p* = 0.024), more extraversion (NEO, *β* = 0.123, *CI* = −0.044–0.206, *p* = 0.002), more drinking at baseline (ESPAD, *β* = 0.283, *CI* = 0.200–0.359, *p* = 0.000), and stronger ventral striatal activation during reward anticipation (MID, *β* = 0.132, *CI* = 0.042–0.222, *p* = 0.004).

## Discussion

The main aim of the present study was to characterize differential predictors of hazardous alcohol use at age 14 and 16 in participants with and without familial risk.

Taken together, clustering by familial risk of alcohol and illicit drug misuse and subsequent predictor analyses revealed both overlapping and distinct predictor profiles across the two groups. Hopelessness, smoking history, and prenatal exposure to alcohol were associated with baseline hazardous drinking in both groups, while life events only contributed to baseline hazardous drinking in the presence of familial risk only contributed in the absence of familial risk. Subsequent hazardous drinking was predicted by life events, extraversion, and baseline alcohol consumption in both groups, while impulsivity (self-report via TCI-R) only significantly predicted subsequent hazardous drinking in the presence of familial risk. Analyses in the overall sample also emphasize the role of familial risk, as it predicted hazardous drinking at age 14 and closely missed significance in predicting subsequent hazardous drinking.

These findings suggest that depressed mood, hazardous behaviors, and significant life events strongly contribute to early (in our sample, at age 14) hazardous alcohol use, while subsequent use at age 16 is additionally influenced by personality traits such as impulsivity and extraversion, suggesting an effect of peer-related person-environment interactions in adolescents’ development of drinking behavior. At the same time, our findings are in line with recent findings in older adults with a positive family history of drug abuse^26^, we found impulsivity and risk-taking behaviors to be sizeable predictors of increased and harmful alcohol use in adolescents with a positive family history of drug abuse. Thus, the effect of family history on alcohol use in adolescents may be mediated by increased impulsivity and risk-taking behavior in adolescents with familial risk.

Most notably, however, our results suggest that early alterations in the reward system predict hazardous alcohol use at both ages 14 and 16 in participants without familial risk only. While this is in line with previous findings showing no association between ventral striatal activation and family history in adolescents^19^, the finding also suggests that in adolescents without familial risk but increased alcohol use, alterations in the reward system may emerge or co-occur with increased alcohol use. Such patterns have previously been shown for heavy drinkers in late adolescence/early adulthood, and conspicuously did not show any association with familial risk^47^. Therefore, striatal alterations could have presented as marker for increased alcohol use and hence increased risk in adolescents without a family history of drug abuse.

Given that adult participants with a family history seem to be more susceptible to hyperactive reward systems, possibly in conjunction with altered levels of impulsivity^20^, the finding of a differentially predictive effect of neurobiological alterations in the reward system at ages 14 and 16 in adolescents without a significant family history suggests that, in the absence of familial risk, early hypersensitivity of the reward system may be a strong moderator of hazardous alcohol use. The effect sizes we find in this sample suggest that alterations in the reward system may account for roughly 2% of the variance in hazardous drinking in adolescents, while overall models including all predictor variables explained up to 22% of the variance.

Compared to previous studies, the comparably sizeable amount of variance explained in our subcluster analyses underlines the potential for subgroup or cluster-based, more individualized prediction approaches for hazardous alcohol use in adolescents. Results suggest that individual differences in personality, cognition, life events, brain function, and drinking behavior contribute differentially to the prediction of future alcohol misuse. However, our key finding highlights that beyond personality and life event variables, in the absence of familial risk, neurobiological alterations in adolescents’ reward systems are directly associated with future substance use disorder severity. This approach may inform more individualized future preventive interventions.
